# Cross-talk among metabolic parameters, esophageal microbiota, and host gene expression following chronic exposure to an obesogenic diet

**DOI:** 10.1038/srep45753

**Published:** 2017-03-31

**Authors:** Nadeem O. Kaakoush, Virginie Lecomte, Christopher A. Maloney, Margaret J. Morris

**Affiliations:** 1 School of Medical Sciences, UNSW Australia, Sydney 2052, NSW, Australia

## Abstract

Unhealthy diets, and ensuing weight gain, predispose individuals to the development of esophageal adenocarcinoma. We examined the effect of chronic high fat diet (HFD) on the esophageal microbiota of Sprague Dawley rats using Illumina MiSeq amplicon sequencing (V4, 515 F/806 R) and on esophageal expression of *IL18, PTGS2, PPARA, FFAR3*, and *CRAT*. The relationships among metabolic parameters, esophageal microbiota, and host gene expression were determined. We observed a significant difference between the upper and lower esophageal microbiota in control fed rats, emphasized by enrichment of *Lactobacillus* species in the lower esophagus. Rats on HFD gained significantly more fat and had reduced insulin sensitivity. Diet type significantly affected the esophageal microbiota, with *Clostridium* sensu stricto being enriched in both upper and lower segments of HFD fed rats. Of interest, bacterial pathways related to carotenoid biosynthesis were significantly decreased in the lower esophagus of HFD fed rats. We observed strong correlations between metabolic parameters, the esophageal microbial profiles, and host esophageal gene expression. In particular, *Fusobacterium, Rothia*, and *Granulicatella* showed consistent correlations across a range of metabolic and gene markers. Our data indicates that unhealthy diets can significantly alter the esophageal microbiota, and enrich for bacterial species previously associated with chronic gastrointestinal diseases.

Obesity is a multifactorial disease that represents a significant and rising burden on human health. Genetic susceptibility, relative malnutrition, and the composition and metabolic contribution of the intestinal microbiome all play compounding roles in the development of obesity^1^. In particular, the strong impact unhealthy diets have on the composition of the intestinal microbiota and its capacity to harvest energy from different foods^2^,^3^,^4^, is now considered one of the main etiological factors of obesity.

Being overweight or obese can predispose individuals to a number of diseases with high mortality, including esophageal adenocarcinoma (EAC)^1^,^5^. One of the underlying factors responsible for the increased risk of EAC in individuals with high Body Mass Index (BMI) is increased prevalence of reflux symptoms^5^. This in turn leads to these individuals developing gastro-esophageal reflux disease (GERD), which could place them in the early stages of the EAC cascade and at risk of progressing to Barrett’s esophagus (BE), a protective shift in the esophageal lining towards an intestinal-like phenotype. The increased reflux symptoms can be related to specific dietary factors (e.g. high fat foods) or physical factors such as hiatal hernias and increased abdominal obesity^5^. For example, a dietary pattern of high meat/fat intake, in particular high-fat dairy foods, has been associated with increased risk of EAC^6^. Further, higher levels of EAC have been observed in the Levrat rat reflux model following a high-fat diet (HFD) as compared to low-soybean oil diet^7^.

There is accumulating evidence that the esophageal microbiota is involved in the EAC cascade at different stages. Examples of this include the relaxation of the gastric sphincter following exposure to lipopolysaccharide being associated with increased reflux, and the association of certain microbial species with the expression of genes involved in the innate immune response in the esophagus^8^. Despite this, our understanding of the relationship between unhealthy diets, higher BMIs, and the esophageal microbiota is limited to the identification, using denaturing gradient gel electrophoresis, of a decrease in *Lactobacillus* abundance in the distal oesophagus of Sprague-Dawley rats fed a HFD^9^.

Given the lack of information surrounding the underlying mechanisms for the link between obesity and esophageal disease, in this study we examined metabolic parameters, esophageal microbiota and host esophageal expression of a range of markers involved in inflammation and fatty acid metabolism of two groups of rats fed control or a HFD for 16 weeks, and determined the associations between these factors.

## Results

### Rats fed a high fat diet gained more fat and developed insulin resistance

**Figure 1:**
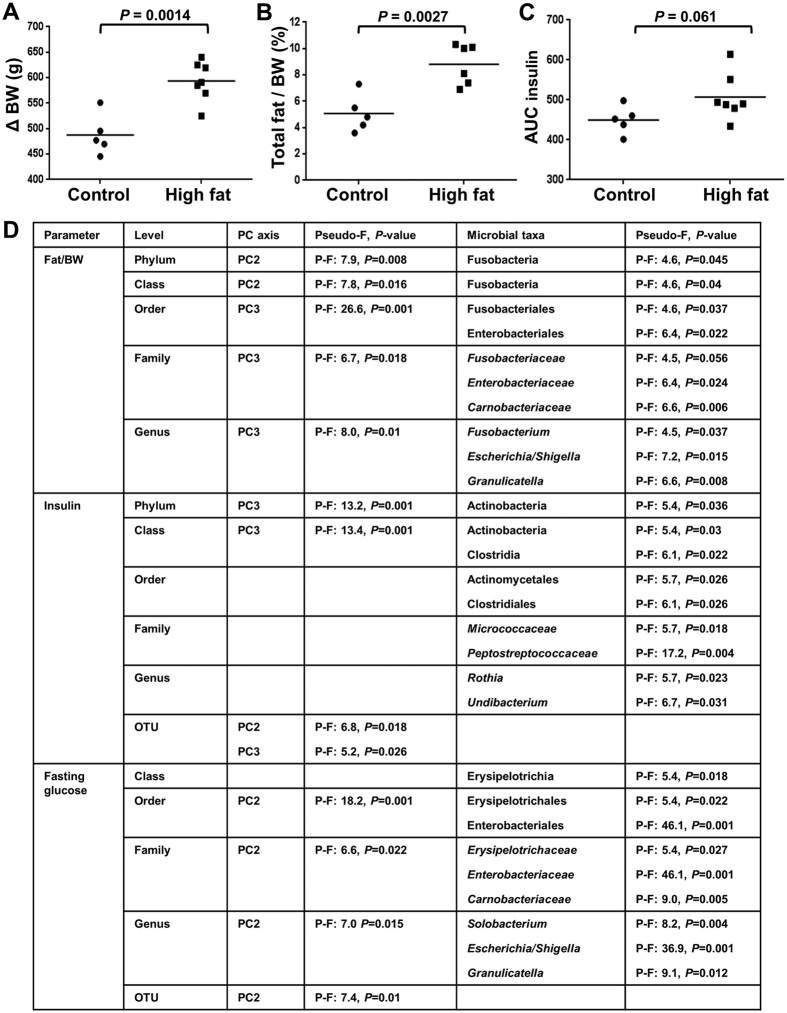
Metabolic parameters of rats fed either control or high fat diet. (**A**) Rats on a HFD (n = 7) had a significant increase in body weight compared to rats on control diet (n = 5). (**B**) Percentage of fat relative to body weight was significantly higher in rats fed HFD compared to controls. Total fat (g): Control: 27.8 ± 4.6; High fat: 57.8 ± 4.2; n = 12; *P* = 0.0010. (**C**) Rats on HFD had a higher area under the curve on the insulin tolerance test compared to controls. (**D**) Correlation of metabolic parameters (resemblance using Euclidean distance) with microbial principal components and relative abundances at different taxonomic levels using DistLM analysis. Correlations with relative abundances of OTUs were not performed. BW: Body weight, AUC: Area under the curve, P-F: Pseudo-F.

HFD fed rats gained more weight than controls, and at the end of the experiment (16 weeks of diet), HFD fed rats were significantly heavier than control fed rats (Delta Body Weight: Control: 487.7 ± 17.8 g; HFD: 593.6 ± 14.8 g; P = 0.0014; Fig. 1A). Consistently, total fat and percentage fat relative to body weight were both significantly higher in HFD fed rats as compared to control fed rats (Fig. 1B). Fasting blood glucose concentrations were significantly different between the two groups at 14 weeks of diet (Fasting glucose (mM): Control: 5.22 ± 0.21; HFD: 5.87 ± 0.10; *P* = 0.030). The glucose tolerance test conducted immediately after showed that the HFD fed rats had a higher area under the curve (AUC) as compared to the chow fed rats (Glucose tolerance test (AUC_glucose_): Chow: 2052 ± 153; High fat: 2514 ± 83 mM.min; *P* = 0.066), indicating reduced glucose tolerance in HFD fed rats compared to controls. An insulin tolerance test conducted at 15 weeks of diet showed an increased AUC (*P* = 0.061) in HFD fed rats as compared to control fed rats (Fig. 1C), which was suggestive of reduced insulin sensitivity in the HFD fed rats.

One control fed rat that gained a comparable amount of weight to HFD fed rats was considered an outlier and excluded from the above analyses (Supplementary file 1). While this outlier control rat had a slightly higher percentage fat relative to body weight as compared to the average of other control rats, it was lower than the average of HFD rats (Fig. 1B, Supplementary file 1). Further, glucose and insulin measurements were comparable to other control rats (Supplementary file 1).

### The esophageal microbiota differs by location and diet

**Figure 2:**
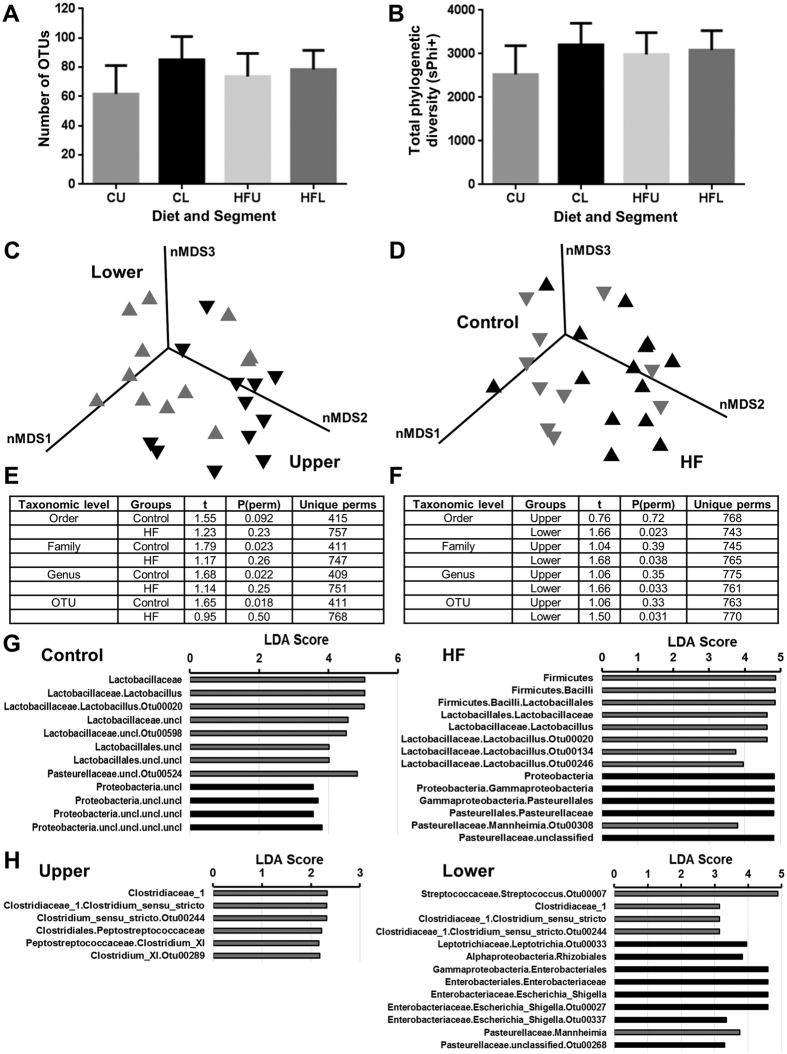
Analysis of the upper and lower esophageal microbiota of rats fed either control (n = 5) or high fat diet (n = 7). (**A**) Number of OTUs identified across different esophageal segments and diets. High variability was observed in the microbial diversity of the esophagus, with a trend towards higher diversity in the lower esophagus. A similar pattern was observed for Shannon’s diversity. Errors are presented as SEM. (**B**) Total phylogenetic diversity calculated across different esophageal segments and diets. Errors are presented as SEM. (**C**) Non-metric multidimensional scaling (nMDS) plot following square root transformation and Bray-Curtis resemblance of relative abundance at genus level shows small separation between the upper and lower esophagus of rats. Both diet groups were present which contributed to some of the observed variability. Upper: Black inverted triangle; Lower: Gray triangle. (**D**) nMDS plot with similar parameters shows minor separation in the esophageal microbiota of rats fed control or HFD. Both lower and upper segments were present which contributed to some of the observed variability. HFD: Black triangle; Control: Gray inverted triangle. (**E**) Comparison of the esophageal microbiota between upper and lower segments at all taxonomic levels using PERMANOVA in each of the control and HFD rat groups. No tests at phylum or class level were significant. High variability in HFU samples contributed to lack of significance across corresponding tests. (**F**) Comparison of the esophageal microbiota between control and HFD rats at all taxonomic levels using PERMANOVA in each of the upper and lower segments. No tests at phylum or class level were significant. High variability in HFU samples contributed to lack of significance across corresponding tests. (**G**) LEfSe analysis identifies microbial taxa differentially abundant (LDA Score >2.0, *P* < 0.05) between upper (black) and lower (gray) esophagus. Control and HFD rats were analyzed separately. (**H**) LEfSe analysis identifies microbial taxa differentially abundant (LDA Score >2.0, *P* < 0.05) between control (black) and HFD (gray) fed rats. Upper and lower segments were analyzed separately.

The esophageal microbiota was analyzed across two different factors, segment location (upper and lower) and diet (control and HFD). No significant differences in α-diversity (number of OTUs, species evenness, species richness, or Shannon’s diversity, Fig. 2A) or phylogenetic diversity (Fig. 2B) were observed across both factors. This was due to high variability in diversity measures observed across samples in all sub-groups (CU: control upper, CL: control lower, HFU: high fat upper, HFL: high fat lower).

nMDS plots indicated that some separation across location can be observed (Fig. 2C, Supplementary file 1), and this was confirmed using PERMANOVA (Fig. 2E). Specifically, significant differences in the microbiota composition of the upper and lower esophagus in control rats were found at the family, genus and operational taxonomic unit (OTU) levels (Fig. 2E). In contrast, no significant differences in microbial composition were detected between the upper and lower esophagus of HFD rats. LEfSe analysis was employed to determine if specific differences were observed across location (Fig. 2G). *Lactobacillus* taxa were consistently found to be enriched in the lower esophagus as compared to the upper esophagus in each of the control fed and HFD fed rats (Relative abundance: CU: 3.58%, CL: 25.08%, HFU: 2.15%, HFL: 12.86%; Fig. 2G).

nMDS plots identified some separation across diet (Fig. 2D, Supplementary file 1), and significant differences (PERMANOVA) were found in the lower esophageal microbiota between control and HFD fed rats at the order, family, genus and OTU levels (Fig. 2F). LEfSe analysis was then employed to determine if specific differences were observed across diet (Fig. 2H). *Clostridium* sensu stricto (OTU244) was found to be significantly more abundant in HFD fed rats as compared to control fed rats in both the upper and lower esophagus (Fig. 2H). The consensus 16 S rRNA gene sequence of OTU244 had 99% similarity to *Clostridium celatum*. Interestingly, the abundance of *Escherichia/Shigella* was found to be decreased in the HFD-fed rats but only in the lower esophagus (Fig. 2H). Further, while not identified using LEfSe, there appeared to be a decrease in abundance of *Lactobacillus* in the lower esophagus of HFD-fed rats (CL: 25.08%, HFL: 12.86%).

Of interest, the upper and lower esophageal microbiota of the outlier control rat were comparable to other control rats (Supplementary file 1).

### Composition of the esophageal microbiota correlates with metabolic parameters

The associations between the esophageal microbiota and anthropometric and metabolic parameters were analyzed using distance-based linear modeling (both upper and lower segments). Consistent correlations were observed between the percentage fat relative to body weight and the microbial principal components across taxonomic levels (Fig. 1D). Further, strong correlations between fasting glucose and insulin sensitivity and the microbial principal components were also identified across different taxonomic levels (Fig. 1D). Distance-based linear modelling (DistLM) was also applied to determine the specific microbial taxa that correlated with these measurements (Fig. 1D). *Granulicatella* and *Escherichia/Shigella* (both decreased in relative abundance in HFD) correlated with both the percentage fat relative to body weight and fasting glucose levels. *Solobacterium (Erysipelotrichaceae*) and *Rothia (Micrococcaceae*, increased in relative abundance in HFD) correlated with fasting glucose and insulin, respectively. Of particular interest, *Fusobacterium* correlated with percentage fat relative to body weight (Fig. 1D), with increased relative abundance in rats with higher ratios. In support of this, the percentage relative abundance of *Fusobacterium* was higher in HFD-fed compared to control-fed rats (CU: 3.52%, CL: 2.56%, HFU: 6.31%, HFL: 4.22%). All correlations showed consistent results with and without the control outlier rat.

### Metabolic contributions of the esophageal microbiota differs by location and diet

**Figure 3:**
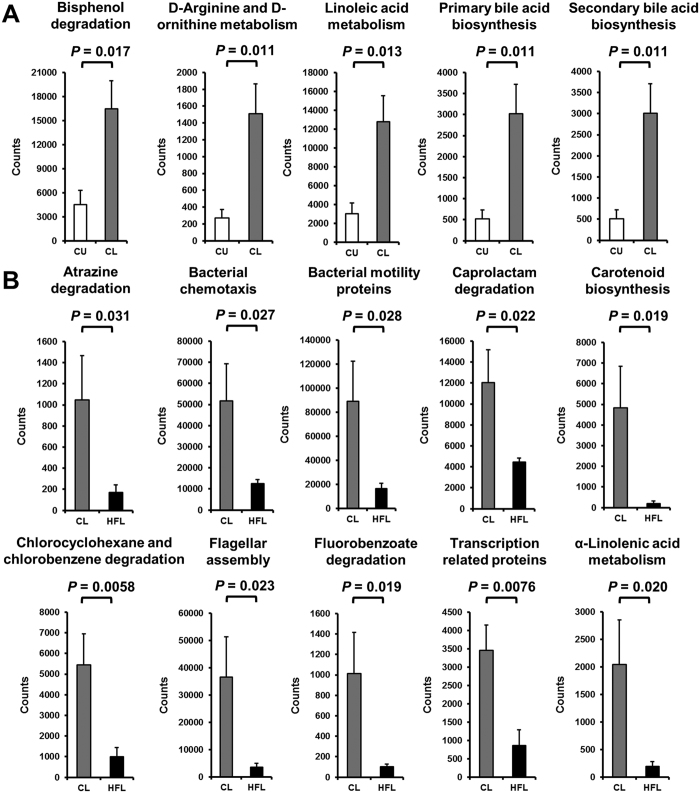
Prediction of the metabolic contributions of the esophageal microbiota using PICRUSt. (**A**) Pathways that were significantly different between the upper and lower segments. *P*-values are FDR adjusted. No pathways were found to be significantly different between HFU and HFL after FDR correction. (**B**) Pathways that were significantly different between control and HFD rats. *P*-values are FDR adjusted. No pathways were found to be significantly different between CU and HFU after FDR correction. High variability in HFU samples contributed to lack of significance across both tests.

Microbial metabolic contributions were predicted from the 16 S rRNA gene data using PICRUSt, and compared across diet and segment location. Following correction for false discovery, five pathways were found to be significantly differentially abundant in the lower esophagus as compared to the upper esophagus of control rats (Fig. 3A). These included pathways associated with bile acid biosynthesis and linoleic acid metabolism (Fig. 3A). On the other hand, ten pathways were significantly different in the lower esophagus of HFD fed rats as compared to the lower esophagus of control fed rats (Fig. 3B). Among these pathways, carotenoid biosynthesis, α-linolenic acid metabolism, and three pathways related to bacterial motility were all diminished. These results were consistent with the identification of significant differences in microbial composition (PERMANOVA) between the upper and lower esophagus only in control rats (up to taxonomic level Family) and between control and HFD rats only in the lower esophagus (up to taxonomic level Order).

### Expression of host genes correlate with metabolic parameters and microbial profiles

**Figure 4:**
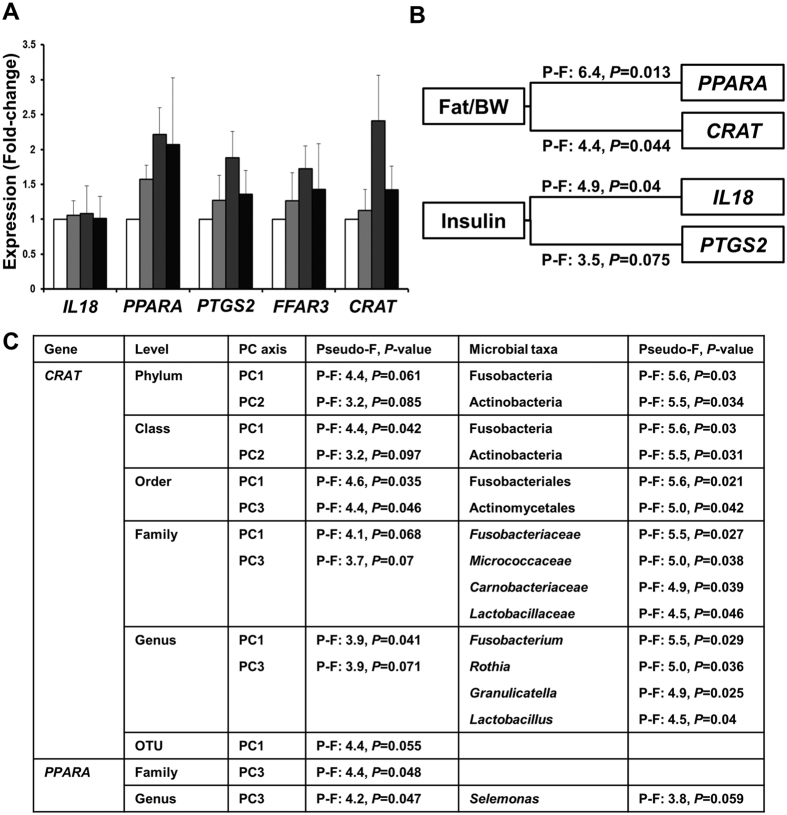
Host gene expression across different esophageal segments and diets and its relationship with metabolic parameters and the esophageal microbiota. (**A**) Gene expression of *IL18, PPARA, PTGS2, FFAR3*, and *CRAT*. Upper segments of control rats (CU) were employed as the reference group. CU: White (n = 6); CL: Light gray (n = 6); HFU: Dark gray (n = 7); HFL: Black (n = 7). (**B**) Association of gene expression with metabolic parameters using DistLM analysis. (**C**) Correlation of metabolic parameters (resemblance using Euclidean distance) with microbial principal components and relative abundances at different taxonomic levels using DistLM analysis. Correlations with relative abundances of OTUs were not performed. Consistently, square root transformation and Bray-Curtis resemblance of relative abundance at different taxonomic levels correlates with CRAT expression (Phylum: Pseudo-F: 3.7, *P* = 0.037; Class: Pseudo-F: 3.1, *P* = 0.029; Order: Pseudo-F: 2.3, *P* = 0.069; Family: Pseudo-F: 2.3, *P* = 0.047; Genus: Pseudo-F: 2.1, *P* = 0.064; OTU: Pseudo-F: 1.7, *P* = 0.096). BW: Body weight, P-F: Pseudo-F.

The expression of five genes involved in inflammatory processes related to EAC (*IL18* and *PTGS2*) or in fatty acid metabolism (*PPARA, FFAR3*, and *CRAT*) were measured in all samples. While a number of genes showed upregulation in the lower esophagus compared to the upper esophagus (*PPARA*) and in HFD fed rats compared to control rats (*PPARA, PTGS2*, and *CRAT*) (Fig. 4A), no comparisons reached significance due to the high variability present across the subgroups. Associations between the expression of these five genes and metabolic parameters were examined, and significant correlations between genes involved in fatty acid metabolism (*PPARA* and *CRAT*) and percentage fat relative to body weight were identified (Fig. 4B). Further, significant correlations between genes involved in inflammatory processes (*IL18* and *PTGS2*) and insulin sensitivity were also observed (Fig. 4B).

The relationships between the expression of these genes and the microbial profiles were then determined. Consistently, significant correlations between the expression of *CRAT* and *PPARA* and microbial principal components were identified across taxonomic levels (Fig. 4C). Moreover, DistLM analysis showed high concordance between the specific microbial taxa associated with expression of *CRAT (Fusobacterium, Rothia*, and *Granulicatella*) and those associated with the anthropometric and metabolic parameters (Figs. 1D and 4C).

## Discussion

The prevalence of obesity is on the rise worldwide due to the increased consumption of unhealthy diets rich in saturated fats and simple sugars, contributing, in part, to an increase in prevalence of other associated diseases such as adenocarcinomas of the digestive tract^1^,^5^. In fact, an analysis by Olsen *et al*. calculated that high BMI (≥30) accounts for roughly 23% of EAC cases in their cohort^10^. Given that the esophageal microbiome is a possible etiological agent of EAC, and the strong impact that unhealthy diets have on the intestinal microbiome, in this study, the effects of HFD, and ensuing weight gain, on the esophageal microbiota and host gene expression were analyzed. The relationships across these factors were also examined.

An important finding in our study is that the microbiota was not homogenous throughout the esophagus. Significant differences in microbiota composition were found between the upper and lower segments of the esophagus, and these differences could be observed up to the family taxonomic level. This is to be expected as the lower esophagus is more likely to be exposed to the gastric environment, and is interesting given the findings of studies that have reported the microbiota of the lower gastrointestinal tract to vary with location^11^,^12^,^13^. A differentiating factor between the lower and upper esophagus, regardless of diet type, was the increased abundance of *Lactobacillus* in the lower esophagus. The high tolerance of these bacterial species to exposure to acid and bile^14^,^15^ may be a contributing factor to their higher abundance in the lower esophagus. This is supported by the enrichment of bacterial metabolic pathways related to bile acids in the lower esophagus. Interestingly, Zhao and colleagues identified a decrease in the abundance of *Lactobacillus* in HFD rats^9^, and a similar yet non-significant decrease was observed in our study but only in the lower esophagus.

As expected, HFD fed rats gained significantly more weight than those on control diet, and the weight gain corresponded to significantly higher levels of body fat. Further, HFD fed rats had higher fasting glucose levels, reduced glucose tolerance and increased insulin resistance compared to control fed rats at 14–15 weeks of diet. The microbial profiles of the lower esophagus significantly differed between diet types, and these differences were observed up to the order taxonomic level. These results indicate that despite the fact that esophageal food transit occurs quickly, HFD can still significantly impact the microbiota in the esophagus. However, the effect of HFD on the esophageal microbiota does not appear to be as pronounced as has been previously observed for the intestinal microbiota^4^.

One bacterial taxon that was found to be significantly enriched following HFD in both the upper and lower esophagus was *Clostridium* sensu stricto. Bacterial species belonging to this genus are known producers of short chain fatty acids (SCFA) such as acetic acid and n-butyric acid, but also other organic acids such as succinic and lactic acid^16^,^17^,^18^. Little is known about the bacterial species with highest similarity to that enriched in the HFD-modified esophagus, *Clostridium celatum*; however, an early study that isolated this bacterium from human feces reported that it produces acetic and formic acid^19^. We have recently hypothesized that microbial SCFA production may contribute to the transition from squamous to intestinal-like cells seen during the early stages of the EAC cascade^1^. Thus, it would be of interest to determine if SCFA levels are higher in the esophagus of HFD fed rats, and how this may impact the esophageal microenvironment.

Consistent correlations were observed across metabolic parameters, the composition of the esophageal microbiota, and host gene expression. Genes involved in inflammatory processes were found to be correlated with insulin resistance. Given that insulin resistance is believed to have a strong immunological component^20^, the expression of inflammatory genes in the esophagus may in part be a reflection of systemic inflammation in the HFD rats. This is supported by the fact that the levels of these genes were not correlated with microbial profiles (principal components). However, this does not exclude the possibility that individual microbial species may contribute to inflammation. Further, genes involved in fatty acid transport and metabolism were correlated with the levels of percent fat relative to body weight, emphasizing the impact that HFD has on the host either directly or indirectly through modulating the microbiome (e.g. significant decrease in microbial α-linolenic acid metabolism in HFD rats). Strikingly, the same microbial taxa (*Fusobacterium, Granulicatella, Rothia*, and *Escherichia/Shigella*) were implicated across comparisons with metabolic markers and host gene expression, highlighting their potential importance in the esophagus exposed to HFD. The consistency of the correlations with *Escherichia/Shigella* would have likely arisen from the large drop in relative abundance of this bacterial taxon following HFD. The impact of this drop on the esophageal tissue remains to be seen given that this taxon encompasses both commensals and pathogens. However, given that some members of *Escherichia/Shigella* are attracted to increased availability of nitrates due to inflammation^21^, it is important to clarify this finding in the future. In contrast, the increased abundance of *Fusobacterium* species in the esophagus of HFD rats is somewhat alarming, given that these bacteria are not only SCFA producers^22^, but they have also been associated with a range of chronic diseases including oral disease, appendicitis, inflammatory bowel diseases, colon cancer, and EAC^8^,^21^,^23^,^24^,^25^. In addition to *Fusobacterium, Rothia* is a member of the oral microbiota whose levels have been associated with oral disease^21^,^26^. The consequences on the host of an esophageal microbiota rich in *Rothia* and *Fusobacterium* species should be examined in the future in the context of increased inflammation and histological changes observed in the EAC cascade.

The predicted esophageal bacterial metagenome (total genomic information) of the rats showed that carotenoid biosynthesis counts were significantly decreased in the lower esophagus of HFD fed rats when compared to control rats. Studies have shown that carotenoids have antioxidant, anti-apoptotic, and anti-inflammatory properties^27^. In the esophagus, long-term β-carotene therapy has been shown to be beneficial in both patients with GERD and BE^28^. Further, epidemiological studies have found that an increased intake of β-carotene was associated with a decreased risk of BE^6^,^29^. However, of note, our results are derived from predictions and need to be confirmed experimentally.

This study is not without limitations. The number of rats per group is low due to loss of samples and the presence of an outlier, which may have led to variability in the observed changes in host gene expression and microbiota due to diet and segment location. The study would have benefited from histological examination of the upper and lower esophageal tissue, and correlation of any observed changes with the microbiota profiles. Further, it is not clear if our data from the rat esophagus can be translated to humans.

In conclusion, we characterized the esophageal microbiota of rats fed two types of diets, and identified changes that correlated with HFD consumption. These changes were apparent in the lower esophagus, an area that is more susceptible to development of premalignant conditions associated with EAC. Moreover, we identified a striking interplay among host metabolic parameters, the esophageal microbiota and host gene expression. Specifically, the increased abundance of *Fusobacterium* relative to higher adiposity and potential changes in bacterial carotenoid conversion are important avenues of future research.

## Methods

### Rat cohort

Three week-old male Sprague Dawley rats from the Animal Research Centre (ARC, Perth, Australia) were housed in a clean facility, 2 per cage under a 12:12 h light/dark cycle. Upon arrival, rats were split into two groups of equal average body weight and standard deviation (n = 12/12). Control rats were fed an in-house made semi-pure control diet based on AIN 93 M (15.1 kJ/g, 9.1% fat, 14.8% protein, 76% carbohydrate as energy). HFD rats were fed an in-house made semi-pure HFD based on the obesogenic diet SF03-020 (20.4 kJ/g, 43% fat, 17% protein, 40% carbohydrate as energy) for 16 weeks (see Supplementary file 1 for further information on exact diet composition). Esophageal samples could only be collected from 6 control- and 7 HFD-fed rats. All animal procedures were approved by the UNSW Australia Animal Care & Ethics Committee (ACEC 14/44B). All methods were carried out in accordance with the relevant guidelines and regulations.

### Measurement of metabolic parameters

A glucose tolerance test was performed at 17 weeks of age (14 weeks of diet) following an overnight fast. Two g of glucose/kg body weight (50% glucose injectable solution, Phebra Lane Cove West NSW Australia) was administered intraperitoneally to each rat and blood glucose concentrations were measured at 0, 15, 30, 45, 60, 90, and 120 min using an Accu-check Performa glucose meter (Roche diagnostic, Castle Hill, NSW, Australia). Blood samples for insulin measurement were collected in EDTA coated tubes at 0, 15, 30, 60 and 120 min. Plasma samples obtained after blood centrifugation were stored at −20 °C. An insulin tolerance test was performed one week later, 6 hours after food removal. One IU/Kg body weight of Insulin (100IU/mL Actrapid, NovoNordisk) was administered and blood glucose concentrations were measured at 0, 15, 30, 45, 60, 90, and 120 min.

### Sample collection

After 16 weeks of diet, overnight fasted rats (19 weeks old) were anesthetized (xylazine/ketamine 15/100 mg/kg; Provet, Castle Hill, NSW, Australia). After measurement of body weight and naso-anal length, blood was collected in EDTA coated tubes following cardiac puncture. Rats were killed by decapitation. Retroperitoneal and epidydimal white adipose tissues were dissected and weighed (estimated measure of total fat). Two esophageal samples per animal were harvested (lower sample: ~1 cm in length starting from the gastric sphincter; upper: remaining esophageal sample) and stored at −80 °C prior to analysis.

Anthropometric and metabolic parameters were analysed by Student’s two-tailed t-test after verification of the normality and data were transformed as required.

### Microbiota analysis

DNA extraction was performed using the Puregene Core Kit A (Qiagen) according to the manufacturer’s instructions. The concentration and quality of extracted DNA was assessed using a NanoDrop ND-1000 (Thermo Fisher Scientific). The composition of the microbial communities was assessed by high-throughput sequencing of the 16 S ribosomal RNA gene using Illumina amplicon sequencing (2 × 250 bp chemistry, V4 region, 515 F/806 R primer pair). The sequence data were then analysed using protocols from MOTHUR^30^, which included alignment with the SILVA database (release 123), removal of singletons, chimera checking with UCHIME, and classification against the RDP training set (14_032015). Diversity (number of OTUs, species richness, species evenness, Shannon’s diversity, and phylogenetic diversity) analyses and statistical analyses such as non-metric multidimensional scaling (nMDS) plots, permutational MANOVA (PERMANOVA), and DistLM were performed on the reads (n = 41569 ± 2664 total clean reads/sample) using Primer-E^31^ and GraphPad Prism 6. We utilized PERMANOVA and not ANOSIM due to the presence of interacting or nested factors (diet and segment location) in the study design^32^. Linear Discriminant Analysis Effect Size (LEfSe)^33^ analysis was performed using the Galaxy web application^34^. For PICRUSt^35^ analysis, taxonomic classification was performed against the green genes database (gg_13_5_99), and KEGG pathway count predictions were determined using the PICRUSt tool on Galaxy^34^ following normalization by copy number. Pathway counts were compared across groups using ANOVA with a *post-hoc* Tukey’s test followed by Benjamini–Hochberg false discovery rate correction.

### Measurement of host gene expression using qPCR

RNA was extracted from upper and lower oesophageal samples using the Isolate II RNA extraction kit (Bioline) and cDNA was synthesized from extracted RNA using the SensiFast cDNA synthesis kit (Bioline) according to the manufacturer’s instructions. The concentration and purity of the RNA and cDNA were assessed using a NanoDrop ND-1000. The cDNA mastermix was prepared using the SensiMix SYBR Hi-ROX kit (Bioline) according to the manufacturer’s instructions and performed using a Rotor Gene Q PCR cycler (Qiagen). Primers for interleukin 18 (*IL18*), peroxisome proliferator-activated receptor alpha (*PPARA*), prostaglandin-endoperoxide synthase 2 (*PTGS2*), free fatty acid receptor 3 (*FFAR3*), carnitine acetyltransferase (*CRAT*), peptidylprolyl Isomerase A (*PPIA*), glucose-6-Phosphate Isomerase (*GPI*), and proteasome Subunit Beta 2 (*PSMB2*) are listed in Supplementary file 1.

*IL18* and *PTGS2* were selected as they have been consistently shown through genetic and functional studies to be associated with esophageal carcinogenesis^8^,^36^, and with the esophageal microbiota in the case of *IL18*^37^. Given the documented dysregulation of fatty acid metabolism in obesity^38^ and the predicted changes in bacterial fatty acid metabolism in our rat cohort, *PPARA, FFAR3*, and *CRAT* were selected due to their involvement in fatty acid sensing and metabolism. Cycling conditions were: 50 °C for 2 min, 95 °C for 10 min, 40 cycles of 95 °C for 15 s, 55 °C for 30 s, and 72 °C for 30 s, followed by melting curve analysis to verify PCR specificity. For *FFAR3*, a touchdown protocol of 3 degrees per cycle for 4 cycles was implemented at the annealing stage. Data were analysed using the 2^−ΔΔCT^ method following normalisation against the geometric mean of the reference genes *PPIA, GPI* and *PSMB2*, which were independently validated in our cohort. Two technical replicates were analysed per sample per PCR.

## Additional Information

**How to cite this article:** Kaakoush, N. O. *et al*. Cross-talk among metabolic parameters, esophageal microbiota, and host gene expression following chronic exposure to an obesogenic diet. *Sci. Rep.* **7**, 45753; doi: 10.1038/srep45753 (2017).

**Publisher's note:** Springer Nature remains neutral with regard to jurisdictional claims in published maps and institutional affiliations.

## Supplementary Material

Supplementary File 1
